# Allergens labeling on French processed foods – an Oqali study

**DOI:** 10.1002/fsn3.471

**Published:** 2017-05-05

**Authors:** Charlène Battisti, Amélie Chambefort, Olivier Digaud, Barbara Duplessis, Cécile Perrin, Jean‐Luc Volatier, Julie Gauvreau‐Béziat, Céline Menard

**Affiliations:** ^1^ Anses, French Agency for Food Environmental and Occupational Health Safety 14, rue Pierre et Marie Curie 94701 Maisons‐Alfort France

**Keywords:** Allergen, allergen‐free claims, food allergy, labeling, precautionary statement, processed foods, types of brands

## Abstract

The French Observatory of Food Quality (Oqali) aims at collecting all nutritional data provided on labels of processed foods (nutritional information and composition), at branded products level, in order to follow nutritional labeling changes over time. This study carries out an overview of allergens labeling frequencies by distinguishing allergens used in recipes from those listed on precautionary statements, for the fourteen allergen categories for which labeling is mandatory according to European legislation. 17,309 products were collected, between 2008 and 2012, from 26 food categories. Products were classified per family and type of brand (national brands, retailer brands, entry‐level retailer brands, hard discount, and specialized retailer brands). Allergenic ingredients were identified from ingredients lists and precautionary statements. 73% of the 17,309 products studied contained at least one allergen in their ingredients list and 39% had a precautionary statement for one or more allergens. Milk (53%), gluten (41%), and egg (22%) were the most commonly used allergens in ingredients lists. For precautionary statement, nuts (20%), egg (14%), peanut (13%), soybean (12%), and milk (11%) were the most common allergens listed. Precautionary statement was most frequently found among first‐price products (hard discount and entry‐level retailer brands). National brands seemed to use it less frequently. For all these results, differences depended both on food categories and allergen categories. This study will enable to follow allergens labeling and their use as ingredients over time, particularly by assessing an hypothetical increase in allergens presence in processed food.

## 1 INTRODUCTION

The prevalence of food allergies in French population has been estimated at 3.2% by a 2001 French survey (Kanny et al., 2001). This prevalence is estimated at 3.8% for children and 2% for adults (Guenard‐Bilbault et al., 2012). Currently, complete avoidance is the only efficient treatment for food allergies (AFSSA et Ministère de la santé de la famille et des personnes handicapées, 2002). Accurate information on processed foodstuffs labeling is thus needed. Indeed, accidents related to hidden allergens represent 8.6% of the severe allergic accidents reported by Allergo‐Vigilance network (AFSSA, 2008). These accidents are more often related to either lack of labeling on non prepackaged products or to changes in packaging and/or recipe without labeling revision. It can also be caused by a reading error from an allergic person following a change in the recipe (AFSSA, 2008).

In Europe, the 2003/89/EC directive (European Parliament and Council, 2003) established in 2003 a first list of 12 food ingredients which must be indicated on foodstuffs labels as they are likely to cause adverse reactions in susceptible individuals (gluten, crustacean, egg, fish, peanut, soybean, milk, nuts, celery, mustard, sesame, sulfite, and products thereof). In 2007, this list was supplemented with lupin and mollusks (Commission, 2007). Today, allergen rules have been changed with the EU regulation 1169/2011 (European Parliament and Council, 2011). In particular, substances or products causing allergies or intolerances listed in the regulation shall from now on be emphasized through a typeset that clearly distinguishes them from the rest of the ingredients list.

Generally speaking, allergens for which labeling is mandatory can be different among countries. For instance, in the United States, the Food and Drug Administration (USFDA) developed in 2004 the Food allergen Labeling and Consumer Protection Act (FALCPA) (Congress, 2004). This law identified eight food allergens: milk, egg, wheat, soy, peanut, tree nuts, fish, and crustacean shellfish. By comparison, Australian legislation requires mandatory labeling for peanuts, tree nuts, milk, eggs, sesame, fish, crustacean, soy, and gluten (Zurzolo, Mathai, Koplin, & Allen, 2013).

In Europe, the wording which has to be used for adventitious presence of allergen (unintentional contamination by contact with other products on process line, during storage or shipping) is not regulated. According to the French General Directorate for Competition Policy, Consumer Affairs and Fraud Control (DGCCRF), labeling as “may contain traces of” has to be used as a last resort when the risk assessment cannot be monitored (DGCCRF, 2005). In this respect, the French National Association of Food Industries (ANIA) wrote guidelines in order to reduce adventitious presence of allergens (ANIA, 2005). Furthermore, in Europe, regulated threshold values concerning the smallest dose for allergic reaction don't exist. Then, there are no limit values to establish if the allergen has to be mentioned in precautionary labels. By comparison, Australia developed the Voluntary Incidental Trace Labelling (VITAL), a risk management tool for food industry use to assist them with declaring the possible presence of allergens in their products (Zurzolo et al., 2013).

In 2008, a report of the French Food Safety Agency (AFSSA) on “Food allergies and advisory labeling” (AFSSA, 2008) stated that, considering available data, it was impossible to answer to the French consumers' association for Consumption, Housing and Living Environment (CLCV) questions: “What has been the evolution of the complexity of processed foodstuffs formulation over the past years? What is the impact of this complexity on the frequency of allergies accidents?”. In fact, there were no available databases containing recipes of processed foodstuffs to answer these questions.

The French Observatory of Food Quality (Oqali) has been set up in 2008 by the Ministries in charge of Agriculture, Health and Consumer Affairs. It is implemented both by the French Agency for Food, Environmental and Occupational Health and Safety (Anses) and the French National Institute for Agricultural Research (INRA). This Observatory collects and analyses all nutritional data provided on labels of processed foodstuffs, at branded products level. These analyses enable to follow nutritional labeling changes (nutritional information and composition) in processed foods supply, over time (Goglia et al., 2010; Menard et al., 2012). Therefore, all labeling parameters provided on packaging (nutrition labeling, nutrition and health claims, serving sizes, etc.) are collected. Socio‐economic parameters such as market shares and types of brands (national brands, retailer brands, entry‐level retailer brands, hard‐discount brands, and specialized retailer brands) are also taken into account. With more than 40,000 food items in its database (Menard et al., 2011), almost all types of processed foods are now monitored by Oqali. This work aims at giving an overview of mandatory allergens used in the lists of ingredients of processed foodstuffs and on precautionary statements. This first assess will enable to examine changes in labeling practices and uses of allergens as ingredients, notably by documenting a possible increase in allergen use in processed foodstuffs which is one of the hypothesis for the rising prevalence of food allergies.

## 2 METHODS

17,309 branded processed foodstuffs, divided into 26 food categories, were considered in this study. The following categories of products were examined: Baby food, Bread products, Breakfast cereals, Cakes and biscuits, Canned fruits, Cereal bars, Chocolate products, Cold sauces, Crackers, Delicatessen meat, Dessert mixes, Fresh dairy products and similar, Fresh delicatessen products, Frozen pizzas, Fruit juices and nectars, Fruit purees, compotes and desserts, Hot sauces, Ice creams and sorbets, Infant milk, Jams, Margarins, Processed potato products, Ready‐to‐eat canned meals, Soft drinks, Soups and broths, Syrups. These products were collected between 2008 and 2012 (depending on the food category) on the French market. For each food category, processed foods packages were mostly collected during a short period of time and during the same year. They were mainly collected through partnerships with retailers, trade unions, and food manufacturers that either transmitted their packages or allowed pictures to be taken in supermarkets. All nutritional data labeled on the food packages were then entered and codified in the Oqali database (Menard et al., 2011). Only one package size for each food product was included in the analysis. This was to ensure that frequencies were not biased by products with multiple pack sizes. Within each of the 26 food categories studied, products were classified in several families and per type of brand. Families were defined according to different criteria like sales name or recipe. 5 types of brand were considered. National brands correspond to products distributed nationally under a brand name owned by a food manufacturer. Retailer brands gather products carrying the brand of the retailer rather than the producer and sold only in their own supermarket chain. Entry‐level retailer brand products correspond to first‐price retailer brand products: their plain packaging often reveals this positioning. Hard discount stores sell products at prices lower than the typical market value, with a focus set on price rather than service, display or wide choice. Specialized retailer brands correspond to frozen products sold in freezer centers and by home selling companies.

In order to estimate the market coverage of the processed foodstuffs collected by Oqali, sales volumes data were bought from Kantar Worldpanel (representative household purchases data of French population) in accordance with the years of collection of Oqali samples. Sales volumes data from Kantar Worldpanel were associated to corresponding Oqali products thanks to Kantar Worldpanel descriptors. Market shares could then be calculated for each product at food sector level.

From the ingredients lists of the 17,309 Oqali products considered, allergenic ingredients were identified. Allergen categories studied were the fourteen food ingredients which must be indicated on foodstuffs labels according to the European regulation (Commission, 2007), as they are likely to cause adverse reactions in susceptible individuals: cereals containing gluten (namely wheat, rye, barley, spelt, kamut, or their hybridized strains), crustacean, egg, fish, peanut, soybean (including soya lecithin), milk (including lactose), nuts (namely almonds, hazelnuts, walnuts, cashews, pecan nuts, Brazil nuts, pistachio nuts, macadamia or Queensland nuts), celery, mustard, sesame seeds, sulfite, lupin, and mollusks, including products thereof. In accordance with the regulation, some ingredients or substances derived from listed allergens were not considered as allergenic ingredients (for example wheat‐based maltodextrins or fully refined soybean oil and fat). The frequency analysis of allergens used as ingredients took into account allergens listed in ingredients lists and those mentioned at the end of the list preceded by statement like “contain”. Conversely, the analysis of precautionary statements took into account allergenic ingredients introduced by statement like “may contain traces of”, “manufactured in a facility that also processed” or “may be present”, grouping adventitious presence and traces. If products contained both forms of labeling (listed as used in the product and had precautionary statement for the same allergen category), allergen was only considered as used in the product recipe, so it was not accounted in the estimation of precautionary statements.

Oqali database also enabled to list products with “allergen‐free” claims. Thus, from the labels of the 17,309 Oqali products considered, allergen‐free claims were identified in order to establish frequency of product bearing them. The same allergen categories as mentioned above were studied.

## 3 RESULTS

### 3.1 Food categories and market coverages

The distribution of the 17,309 products collected according to their food category and the estimated market coverages per food category are available in Table 1. The estimated market coverages reached by Oqali samples at food category level varied from 49% (Crackers‐2009) to 89% (Infant milk‐2012).

**Table 1:** Food categories considered in the study, with their associated number of products, year of data collection, and estimated market coverage per total food category

Food category	Year of data collection	Number of foodstuffs taken into account	Estimated market coverage^a^
Baby food	2012	976	88%
Bread products	2009	619	57%
Breakfast cereals	2008	335	75%
Cakes and biscuits	2008	1,692	70%
Canned fruits	2009	184	69%
Cereal bars	2010–2011	170	79%
Chocolate products	2009	750	68%
Cold sauces	2011	500	76%
Crackers	2009	594	49%
Delicatessen meat	2010	1,164	66%
Dessert mixes	2009	155	67%
Fresh dairy products and similar	2008–2009	1,553	66%
Fresh delicatessen products	2008–2009–2010‐2011	1,890	66%
Frozen pizzas	2010	213	62%
Fruit juices and nectars	2009–2010	790	55%
Fruit purees, compotes, and desserts	2009	440	68%
Hot sauces	2010	294	77%
Ice creams and sorbets	2010–2011	1,476	67%
Infant milk	2012	117	89%
Jams	2009	339	65%
Margarins	2011	95	82%
Processed potato products	2011	629	76%
Ready‐to‐eat canned meals	2010	714	71%
Soft drinks	2009–2010	756	78%
Soups and broths	2011	560	77%
Syrups	2009–2010	304	69%
**Total**	**2008–2012**	**17,309**	**70%**

a Sales volumes ratio of products collected by Oqali versus total sales identified by Kantar Worldpanel.

### 3.2 Allergens labeling

Overall, 77% (*n* = 13,322) of the 17,309 products considered mentioned at least one allergen in their ingredients list or in a precautionary statement. Accordingly, 23% (*n* = 3,987) did not mention any allergen, neither in the ingredients list nor in a precautionary statement.

More specifically, 73% (*n* = 12,722) of the branded foodstuffs collected contained at least one allergen in their ingredients list and 39% (*n* = 6,762) had a precautionary statement for one or more allergens (Table 2). These percentages depended on food categories. For instance, the food categories with the highest use of precautionary statement (for one or more allergens) were Cereals bars (*n* = 153; 90%), Chocolate products (*n* = 634; 85%), Ice creams and sorbets (*n* = 1,161; 79%), Crackers (*n* = 456; 77%), Breakfast cereals (*n* = 256; 76%), and Cake and biscuits (*n* = 1,279; 76%) (Table 3). The food categories with the lowest use of precautionary statement were Infant milk (*n* = 2; 2%), Soft drinks (*n* = 7; 1%), Fruit purees, compotes, and desserts (*n* = 3; 1%), Fruit juices and nectars (*n* = 4; 1%), Syrups (*n* = 1; 0.3%), and Canned fruits (*n* = 0; 0%) (Table 3).

**Table 2:** Prevalence of labeling for each category of allergen among the 17,309 products surveyed

Allergen category	Presence of the allergen in the ingredients list^a^		Precautionary statement^a^		No declared allergen (neither in the ingredients list nor in a precautionary statement)^a^	
	*n*	%	*n*	%	*n*	%
Milk	9,222	53	1,935	11	6,152	36
Gluten	7,087	41	1,512	9	8,710	50
Egg	3,759	22	2,496	14	1,1054	64
Soybean	3,460	20	2,146	12	11,703	68
Nuts	1,419	8	3,443	20	12,447	72
Peanut	231	1	2,295	13	14,783	85
Celery	1,127	7	1,114	6	15,068	87
Mustard	1,001	6	881	5	15,427	89
Sulfite	1,578	9	177	1	15,554	90
Fish	589	3	688	4	16,032	93
Sesame	178	1	1,028	6	16,103	93
Crustacean	226	1	692	4	16,391	95
Mollusk	109	1	411	2	16,789	97
Lupin	66	0.4	94	0.5	17,149	99.1
**At least 1 allergen**	**12,722**	**73**	**6,762**	**39**	**3,987**	**23**

a Among the 17,309 processed products considered collected between 2008 and 2012.

**Table 3:** Prevalence of precautionary labeling for each category of allergen and per food category among the 17,309 products surveyed

Precautionary statements	Milk		Gluten		Egg		Soybean		Sulfite		Nuts		Celery		Mustard		Fish		Peanut		Crustacean		Sesame		Mollusk		Lupin		At least 1	
	*n*	%	*n*	%	*n*	%	*n*	%	*n*	%	*n*	%	*n*	%	*n*	%	*n*	%	*n*	%	*n*	%	*n*	%	*n*	%	*n*	%	*n*	%
Baby food	35	4	1	0.1	39	4	24	2	2	0.2	17	2	21	2	0	0	0	0	5	1	0	0	18	2	0	0	0	0	106	11
Bread products	209	34	0	0	195	32	213	34	4	1	199	32	4	1	8	1	0	0	110	18	0	0	172	28	0	0	37	6	415	67
Breakfast cereals	145	43	4	1	0	0	127	38	4	1	180	54	0	0	0	0	0	0	225	67	0	0	146	44	0	0	1	0.3	256	76
Cakes and biscuits	207	12	5	0.3	326	19	415	25	7	0.4	987	58	20	1	12	1	0	0	673	40	0	0	263	16	0	0	21	1	1,279	76
Canned fruits	0	0	0	0	0	0	0	0	0	0	0	0	0	0	0	0	0	0	0	0	0	0	0	0	0	0	0	0	0	0
Cereal bars	62	36	3	2	15	9	56	33	40	24	127	75	8	5	6	4	0	0	114	67	0	0	58	34	0	0	1	1	153	90
Chocolate products	171	23	391	52	259	35	66	9	1	0.1	394	53	5	1	5	1	0	0	264	35	0	0	10	1	0	0	0	0	634	85
Cold sauces	55	11	23	5	7	1	2	0.4	0	0	1	0.2	28	6	32	6	16	3	1	0.2	0	0	0	0	1	0.2	0	0	79	16
Crackers	147	25	73	12	109	18	66	11	22	4	211	36	128	22	71	12	38	6	145	24	64	11	115	19	0	0	6	1	456	77
Delicatessen meat	62	5	44	4	83	7	32	3	0	0	58	5	45	4	71	6	0	0	0	0	0	0	1	0.1	0	0	0	0	221	19
Dessert mixes	31	20	24	15	27	17	45	29	4	3	50	32	0	0	0	0	0	0	3	2	0	0	7	5	0	0	0	0	77	50
Fresh dairy products and similar	3	0.2	89	6	6	0.4	58	4	1	0.1	82	5	1	0.1	0	0	0	0.0	20	1.3	0	0	2	0.1	0	0	0	0	181	12
Fresh delicatessen products	186	10	109	6	276	15	261	14	44	2	219	12	361	19	219	12	382	20	45	2	390	21	149	8	259	14	25	1	675	36
Frozen pizzas	0	0	0	0	79	37	72	34	13	6	11	5	62	29	79	37	40	19	52	24	39	18	13	6	26	12	0	0	99	46
Fruit juices and nectars	4	1	0	0	0	0	4	1	0	0	4	1	0	0	0	0	0	0	0	0	0	0	0	0	0	0	0	0	4	1
Fruit purees, compotes, and desserts	1	0.2	2	0.5	0	0	0	0	0	0	0	0	0	0	0	0	0	0	0	0	0	0	0	0	0	0	0	0	3	1
Hot sauces	24	8	18	6	8	3	0	0	0	0	1	0.3	23	8	24	8	1	0.3	0	0	1	0.3	22	7	1	0.3	0	0	45	15
Ice creams and sorbets	208	14	462	31	705	48	481	33	9	1	829	56	0	0	1	0.1	0	0	588	40	0	0	3	0.2	0	0	0	0	1,161	79
Infant milk	1	1	1	1	0	0	0	0	0	0	0	0	0	0	0	0	0	0	0	0	0	0	0	0	0	0	0	0	2	2
Jams	31	9	3	1	3	1	0	0	0	0	44	13	0	0	0	0	0	0	0	0	0	0	0	0	0	0	0	0	68	20
Margarines	4	4	0	0	0	0	0	0	0	0	0	0	0	0	0	0	0	0	0	0	0	0	0	0	0	0	0	0	4	4
Processed potato products	153	24	136	22	58	9	40	6	10	2	5	1	120	19	163	26	6	1	40	6	5	1	3	0.5	4	1	2	0.3	242	38
Ready‐to‐eat canned meals	113	16	47	7	133	19	92	13	8	1	7	1	128	18	131	18	142	20	5	1	134	19	9	1	95	13	0	0	272	38
Soft drinks	0	0	3	0.4	0	0	3	0.4	1	0.1	0	0	0	0	0	0	0	0.0	0	0	0	0	0	0	0	0	0	0	7	1
Soups and broths	83	15	73	13	168	30	89	16	7	1	17	3	160	29	59	11	63	11	5	1	59	11	37	7	25	4	1	0.2	322	58
Syrups	0	0	1	0.3	0	0	0	0	0	0	0	0	0	0	0	0	0	0	0	0	0	0	0	0	0	0	0	0	1	0.3
**Total**	**1,935**	**11**	**1,512**	**9**	**2,496**	**14**	**2,146**	**12**	**177**	1	**3,443**	**20**	**1,114**	**6**	**881**	**5**	**688**	**4**	**2,295**	**13**	**692**	**4**	**1,028**	**6**	**411**	**2**	**94**	**1**		

^a^Among the processed products considered of the food category.

Among the 17,309 products considered, Table 2 shows, per category of allergen and for all food categories, the number and percentage of products either using the allergen in their recipe, displaying a precautionary statement for the allergen, or not mentioning the allergen studied at all (neither in the ingredients list nor in a precautionary label). Data has been sorted by percentage of products with no mention of the allergen studied, in ascending order.

For all food categories, the most common categories of allergens contained in the ingredients lists were milk (*n* = 9,222; 53%), gluten (*n* = 7,087; 41%), egg (*n* = 3,759; 22%), and soybean (*n* = 3,460; 20%). Among the ingredients less frequently used, frequencies of products containing nuts, celery, mustard, and sulfite (between 6 and 9% of products) were higher than those using peanut, sesame, crustacean, and mollusk (around 1%). Lupin was the least used (0.4%). Frequencies were also dependent on food categories: for instance, milk was contained in 100% of Frozen pizzas, whereas Canned fruits and Syrups did not contain any milk (data not shown). Likewise, egg was contained in 69% of Cakes and biscuits, 61% of Cold sauces, and 49% of Fresh delicatessen products. In general way, only Canned fruits did not contain any allergen (data not shown).

Regarding the precautionary statements, the most common categories of allergens were nuts (*n* = 3,443; 20%), egg (*n* = 2,496; 14%), peanut (*n* = 2,295; 13%), soybean (*n* = 2,146; 12%), and milk (*n* = 1,935; 11%). Frequencies varied depending on food categories. For example, 75% of the Cereal bars had a precautionary statement on nuts and 52% of the Chocolate products had a precautionary statement on gluten. Only Canned fruits did not list any allergen precautionary statement. Complete results per food category are available in Table 3.

It should be noted that, for all food categories, the following allergens are more often listed on precautionary statements than contained in ingredients lists: nuts (20% against 8%), peanut (13% against 1%), sesame (6% against 1%), crustacean (4% against 1%), fish (4% against 3%), mollusk (2% against 1%), and lupin (0.5% against 0.4%).

### 3.3 Precautionary statement per type of brand

Precautionary statement frequencies per type of brand are available in Table 4. Regardless of the allergen category, precautionary statement was found on 47% of hard discount products (*n* = 1,323), 42% of entry‐level retailer brands (*n* = 444), 37% of retailer brands (*n* = 2,807), and 33% of national brands (*n* = 1,736). Per allergen category, the highest differences between types of brands were for peanut, nuts, milk, and soybean. Differences between types of brands were also observed for gluten, egg, and sesame. Nevertheless, differences depended both on food categories and allergen categories.

**Table 4:** Prevalence of precautionary statement per type of brand, for each allergen category

Precautionary statement	National brands (*n* = 5,330) *26 food categories over the 26 studied*		Retailer brands (*n* = 7,488) *26 food categories over the 26 studied*		Entry‐level retailer brands (*n* = 1,060) *23 food categories over the 26 studied*		Hard discount (*n* = 2,813) *26 food categories over the 26 studied*	
	*n*	%	*n*	%	*n*	%	*n*	%
Peanut	396	7	1,121	15	135	13	551	20
Celery	290	5	542	7	73	7	182	6
Crustacean	194	4	308	4	48	5	126	4
Nuts	795	15	1,411	19	222	21	686	24
Gluten	296	6	593	8	82	8	298	11
Milk	444	8	826	11	185	17	375	13
Lupin	30	1	48	1			16	1
Mollusk	139	3	179	2	18	2	60	2
Mustard	209	4	454	6	44	4	156	6
Egg	622	12	998	13	137	13	451	16
Fish	183	3	339	5	33	3	116	4
Sesame	307	6	489	7	44	4	180	6
Soybean	385	7	1,004	13	108	10	422	15
Sulfite	13	0.2	87	1	12	1	57	2
At least 1 allergen	1,736	33	2,807	37	444	42	1,323	47

### 3.4 Allergen‐free claims

Overall, five allergen categories were mentioned in allergen‐free claims: gluten, milk (including lactose), peanut, egg, and soybean. The frequency of products bearing allergen‐free claims is very low: 4% (*n* = 674) of the 17,309 products studied. Per allergen category, frequencies were between 0.01% (*n* = 2) for soybean and 3% (*n* = 570) for gluten.

Fourteen food categories over the 26 studied had at least one product bearing gluten‐free claim, like “gluten free” or “naturally gluten free”. The food categories with the highest gluten‐free claim frequencies were Baby food (*n* = 499; 51%), Infant milk (*n* = 11; 9%), and Fruit purees, compotes, and desserts (*n* = 23; 5%). For Baby food, results could be related to the directive 2006/125/EC (Commission, 2006) which indicates that the presence or absence of gluten has to be mentioned if the indicated age from which the product may be used is below 6 months. Frequencies were 1% for Fresh dairy products and similar (*n* = 12), Ice creams and sorbets (*n* = 8), Breakfast cereals (*n* = 3), Canned fruits (*n* = 1), and Cereal bars (*n* = 1). Frequencies of the six others food categories were lower than 0.3%: Hot sauces (*n* = 1), Delicatessen meat (*n* = 3), Processed potato products (*n* = 2), Cakes and biscuits (*n* = 4), Ready‐to‐eat canned meals (*n* = 1), and Fresh delicatessen products (*n* = 1).

Six food categories had at least one product bearing milk‐free or lactose‐free claim. Baby food had the highest frequency: *n* = 140; 14%. 2% of Fresh dairy products and similar had a milk/lactose‐free claim (*n* = 24; corresponding to soya desserts), 2% of Margarins (*n* = 2), 2% of Infant milk (*n* = 2), 1% of Fruit purees, compotes, and desserts (*n* = 3), and 0.1% of Cakes and biscuits (*n* = 2).

Over the four food categories bearing peanut‐free claim, Baby food had as well the highest frequency: *n* = 116; 12%. Then, frequencies were 7% for Processed potato products (*n* = 42), 1% for Margarins (*n* = 1), and 0.1% for Cakes and biscuits (*n* = 1).

Egg‐free claims were identified in two food categories: Baby food (*n* = 87; 9%) and Cakes and biscuits (*n* = 2; 0.1%).

Soybean‐free claims were identified only in Chocolate products (*n* = 2; 0.3%).

Per type of brand, allergen‐free claims were mostly found among national brands. No specialized retailer brand and no entry‐level retailer brand product labeled any allergen‐free claim.

## 4 DISCUSSION

This first study, by considering 17,309 processed foodstuffs collected on the French market between 2008 and 2012, provides an overview of allergens use in processed foodstuffs recipes and precautionary statements, for the fourteen allergens of the European regulation.

More specifically, results showed that the most common allergen categories contained in ingredients lists were milk (*n* = 9,222; 53%), gluten (*n* = 7,087; 41%), and egg (*n* = 3,759; 22%). This is partly due to the fact that these allergens are contained in basic ingredients which are widely used in processed foodstuffs. Soybean was also found in 20% of products (*n* = 3,460), particularly due to the use of soya lecithin. Frequencies were also dependent on the food categories, because of the recipes and basic ingredients used. For instance, 100% of Frozen pizzas contained milk due to the presence of cheese; likewise 66% of the Cold sauces contained mustard, one of the main ingredients of vinaigrettes. By comparison, an Australian study over 1,355 food products, collected in 2011, in supermarkets of Melbourne, showed that the most common categories of allergens contained in the ingredients lists were wheat (66.5%), soy (48.1%), and milk (45.1%) (Zurzolo et al., 2013).

Regarding the precautionary statements, 39% (*n* = 6,762) of the products studied had one or more allergens. Percentages also depended on food categories, for instance, the food categories with the highest use of precautionary statement (for one or more allergens) were Cereals bars (*n* = 153; 90%), Chocolate products (*n* = 634; 85%), Ice creams and sorbets (*n* = 1,161; 79%), Crackers (*n* = 456; 77%), Breakfast cereals (*n* = 256; 76%), and Cakes and biscuits (*n* = 1,279; 76%). Some differences in precautionary statement frequencies were observed with other studies. For instance, a study over 20,241 processed foodstuffs collected in 2006 in the United States (Pieretti, Chung, Pacenza, Slotkin, & Sicherer, 2009) showed that 17% of the products had a precautionary statement for at least one of the eight allergens studied (Congress, 2004): milk, egg, wheat, soy, peanut, tree nuts, fish, and shellfish. Food categories with the highest precautionary statement frequencies were Chocolate candy (54%), Cookies (53%), and Baking mixes (40%). The Australian study discussed earlier (Zurzolo et al., 2013), showed that 65% (*n* = 882 of 1,355 products) of the considered products had a precautionary statement for at least one of the 10 allergens studied: namely peanut, tree nuts, egg, milk, sesame, crustacean, fish, wheat, soy, and lupin.

In this study, the most common allergen categories mentioned on precautionary statements were nuts (*n* = 3,443; 20% of the products surveyed), egg (*n* = 2,496; 14%), peanut (*n* = 2,295; 13%), soybean (*n* = 2,146; 12%), and milk (*n* = 1,935; 11%). Except for peanut and sesame, allergens frequently listed in precautionary statements were allergens frequently used as ingredients. This may be due to adventitious presence and could be related to cross‐contamination, more likely for allergens frequently used as ingredients. For peanut, the highest percentage of products with precautionary statement (13%) associated with the lowest presence in ingredients list (1%) could be explained by the fact that this allergen causes allergic reactions among the most severe ones (Bock, Muñoz‐Furlong, & Sampson, 2007) and that the prevalence of French allergic to peanut is high (Morisset, Moneret‐Vautrin, Kanny, & Network, 2005). These results are consistent with those of the American study (Pieretti et al., 2009) who showed that the most common allergen with a precautionary statement were tree nuts (61%) and peanuts (48%), and the Australian study (Zurzolo et al., 2013) who showed that the most common allergens listed on precautionary statements were tree nuts (36.2%) and peanuts (34.1%) followed by sesame (27.5%) and egg (22.6%).

Overall, it is difficult to compare the labeling frequencies of the allergens used in processed foodstuffs recipes or precautionary statements from these different studies because of different ranges of products and different geographic regions. The food categories defined and the allergens considered are different, which impact the results.

Concerning the analysis of the precautionary statements per type of brand, results showed that it was the most frequently found among first‐price products (hard discount and entry‐level retailer brands), then on retailer brands products. National brands seemed to use precautionary statement less frequently. These differences might be partly due to the fact that the offer of products is different between types of brands (Oqali, 2015a) or because of differences in the monitoring of contamination risk. Nevertheless, differences depended both on food categories and allergen categories. Moreover, it is important to notice that these results are representative of the products considered in this study: numbers of products per food sector are different, every type of brand is not represented among each food sector and the presence of one allergen category in precautionary statement might be expected more or less depending on the food sector.

To conclude, this first study, which takes into account 17,309 proceeds products divided into 26 categories, provides a first overview and will permit to examine changes in labeling practices and uses of allergens as ingredients over time, notably documenting a possible rise in the use of allergens in processed foodstuffs recipes. It could then be extended to new categories followed by Oqali, such as Frozen bakery wares and pastries or Frozen ready‐cooked dishes. Data from this study could also be useful to refine exposure estimation, by merging real occurrence frequencies with concentration data. It will be then interesting to analyze the different wordings used for precautionary statement, for instance, “traces of”, “manufactured in a facility that also processes” or “may be present”. Indeed, in Europe, the wording which has to be used for adventitious presence of allergens and the smallest dose for allergic reaction are not regulated. Regulation might be useful to clarify information and then help allergenic people to evaluate the risk.

All these results were detailed in a report available on the Oqali French website (Oqali, 2015b).

## CONFLICT OF INTEREST

None.
